# Supernumerary kidney incidentally detected on staging CT scan: A rare case report

**DOI:** 10.1016/j.radcr.2025.03.066

**Published:** 2025-04-12

**Authors:** Yaman M. Alahmad, Mohamed Lameir Hussein, Ahmad N․ Al-Ekeer, Akram Twair

**Affiliations:** Department of Clinical Imaging, Hamad Medical Corporation, Doha, Qatar

**Keywords:** Supernumerary kidney, Congenital anomaly, Incidental finding, Case report

## Abstract

Supernumerary kidneys are rare congenital anomalies arising from abnormal renal development, with fewer than 100 cases documented in the literature. A 55-year-old male presented with chronic nonbloody diarrhea and was diagnosed with a locally advanced rectal tumor. A staging CT scan incidentally revealed a right-sided supernumerary kidney measuring 4.5 cm, alongside a larger right kidney (7.3 cm) and a normal left kidney (10.2 cm), with independent arterial supply and normal excretory function. While this finding did not impact the patient's oncological treatment plan, awareness of such anomalies is essential for surgical planning to avoid complications. This case underscores the importance of recognizing supernumerary kidneys to guide clinical decision-making in oncological and urological interventions.

## Background

Supernumerary kidneys are rare congenital anomalies resulting from abnormal development during early kidney formation [1]. Normally, kidneys develop through interactions between the ureteric bud and the metanephric mesoderm. In cases of a supernumerary kidney, an additional ureteric bud develops, leading to the formation of an extra kidney [1]. The additional kidney may have its own blood supply, ureter, and independent excretory function.

Fewer than 100 cases of supernumerary kidneys have been reported in the literature [2]. While they are often asymptomatic and discovered incidentally, their presence can complicate surgical planning, particularly in oncological cases [3]. Herein, we report a unique case of an incidentally discovered supernumerary kidney, discussing the clinical significance of such finding.

## Case presentation

A 55-year-old male presented with a 1-month history of chronic nonbloody diarrhea. His medical history was significant for hypothyroidism and a prior open cholecystectomy. Physical examination findings were unremarkable. Colonoscopy revealed a rectal lesion located 8–10 cm from the anal verge. Pelvic MRI revealed circumferential rectal wall thickening, consistent with a locally advanced tumor and regional lymphadenopathy.

**Fig. 1:**
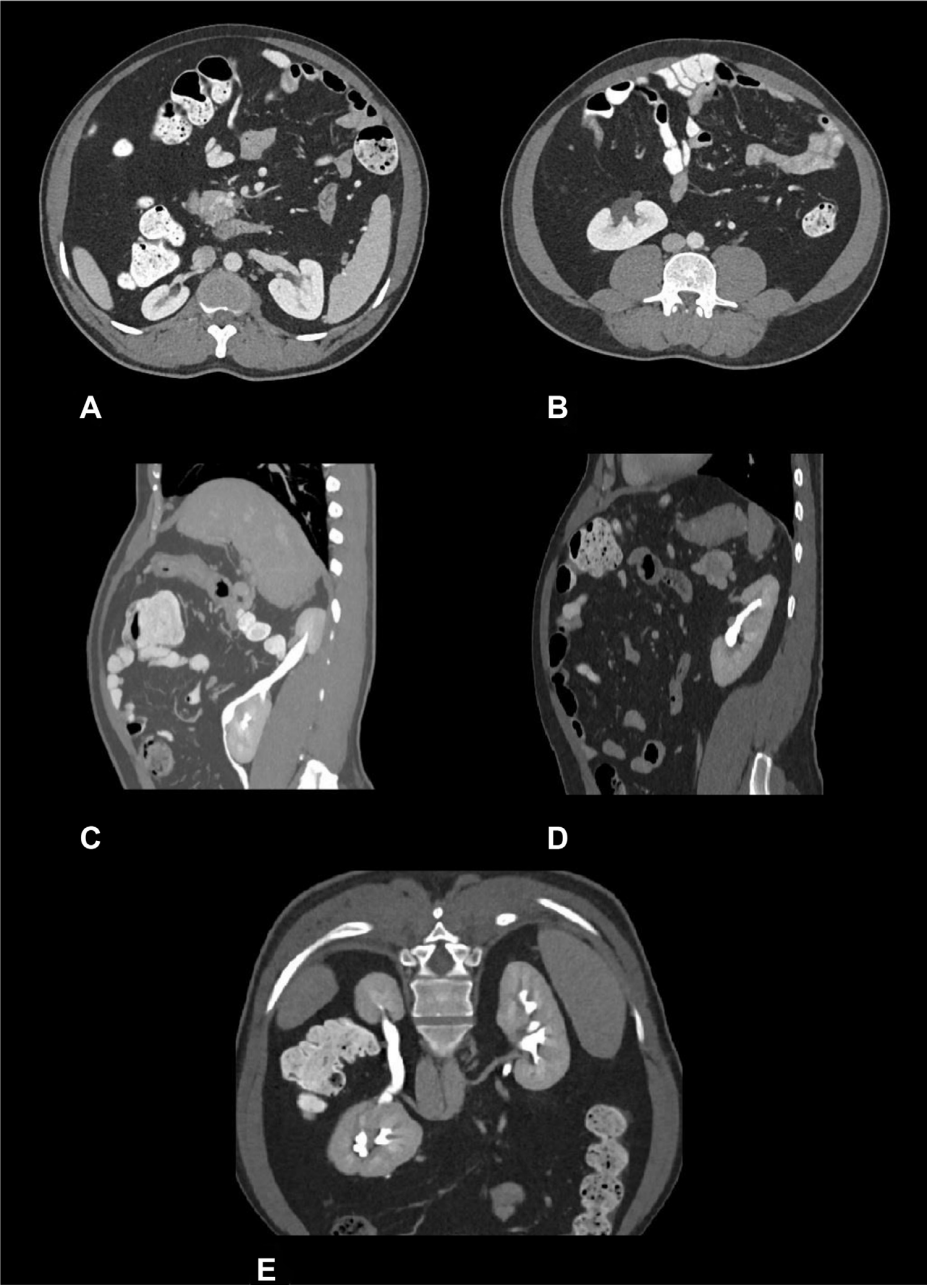
Selected reconstruction images of CT abdomen and pelvis study with intravenous venous acquired contrast. (A and b) show on axial view the 3 kidneys on the abdominal region. (C) Demonstrates on sagittal view the 2 kidneys on the right hemiabdomen, the cranial kidney is smaller, and (D) shows a native left kidney. (E) Coronal view of CT abdomen shows the 3 kidneys.

**Fig. 2:**
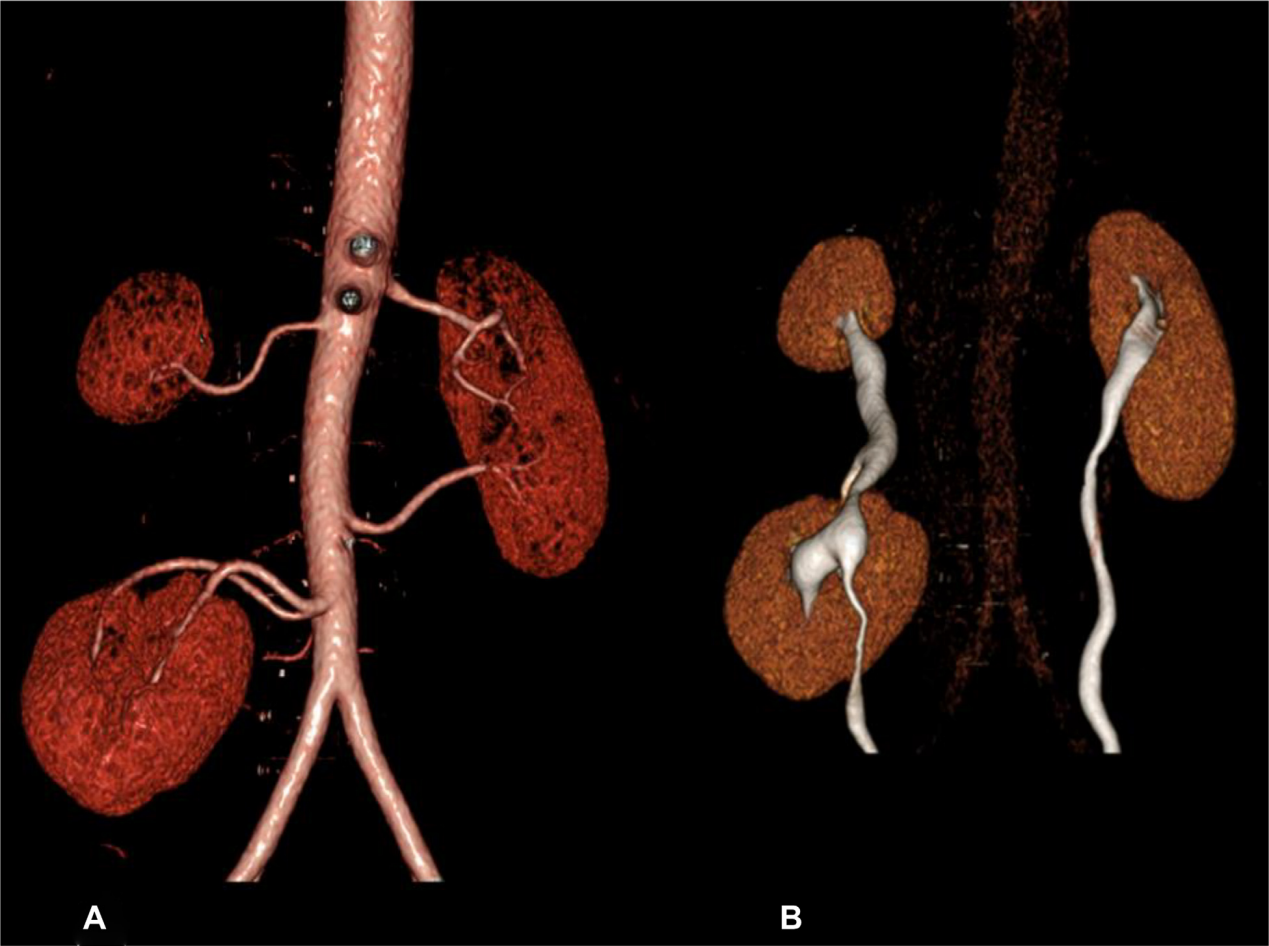
(A) Volume rendering image of CT abdomen and pelvis study demonstrates the arterial supply for the 3 kidneys. The native right kidney, which is caudally placed, is supplied by early bifurcated renal artery arising at lower margin of L3 vertebral body. The supernumerary kidney, which is cranially placed, is separately supplied by a renal artery arising at the level of L1 vertebra. The left kidney is supplied by 2 renal arteries (B) Volume rendering image shows the 3 kidneys are excreting symmetrically with the right superiorly located smaller kidney's (supernumerary) ureter joining the right collecting system at the pelvic-ureteric junction. A single ureter is then draining into the urinary bladder. Note the right native kidney has malrotated pelvis facing anteriorly.

Staging computed tomography (CT) scan of the abdomen and pelvis revealed 2 kidneys of different sizes on the right hemi-abdomen (measured on longest axis 4.5 cm and 7.3 cm for the cranial and caudal located kidneys, respectively) and a single kidney on the left hemi-abdomen (measured 10.2 cm on longest axis) . This finding is indicative of a small, right-sided, cranially located supernumerary kidney with normal excretory function (Fig. 1, Fig. 2) . The arterial supply to the 3 kidneys is shown in Fig. 2. The suprarenal glands were unremarkable. There were no symptoms directly attributable to the supernumerary kidney, and its presence did not contribute to the patient's presenting complaint.

## Discussion

The incidental discovery of a supernumerary kidney in this patient presents a noteworthy clinical scenario. The origin of a supernumerary kidney is linked to early embryologic development, particularly involving the migration and development of the mesonephros [4]. During embryogenesis, 3 sequential kidney structures develop: the pronephros, mesonephros, and metanephros. The metanephros ultimately gives rise to the permanent kidneys, while the mesonephros and pronephros regress [4]. Supernumerary kidneys result from anomalies in the budding or branching of the ureteric bud from the mesonephric duct. This abnormal budding can lead to the development of an additional metanephric blastema, forming a separate kidney structure [4].

Supernumerary kidneys are exceedingly rare and often asymptomatic, with fewer than 100 cases reported in the literature [5]. The clinical significance of such findings lies in their potential to complicate abdominal or pelvic surgeries. The additional kidney may alter anatomical landmarks and vascular structures, increasing the complexity of procedures such as tumor resection or lymph node dissection. Most cases are unilateral, and bilateral supernumerary kidneys are even rarer, with only about 5 such cases documented [5]. Supernumerary kidneys are commonly found on the left side of the abdomen and are often associated with abnormalities in the upper urinary or genital tracts [6].

The differentiation between supernumerary and duplex kidney can be challenging. The presence of separate renal capsules, along with independent arterial blood supply, as in this reported case, often hints towards a supernumerary kidney, whereas a shared renal capsule supports a duplex kidney diagnosis [7]. Accurate identification is essential, as each condition may require different urological management approaches.

In this case, the supernumerary kidney, though functionally normal in an asymptomatic patient, could complicate any future surgical interventions related to the rectal tumor. The patient's treatment plan—systemic chemotherapy followed by radiation—was not directly affected by this finding, but awareness of the supernumerary kidney is crucial for future surgical planning, especially if tumor/metastasis resection becomes necessary.

While the primary focus of this case remains the treatment of malignancy, incidental findings like a supernumerary kidney must be carefully considered, particularly when planning future surgical or urological interventions.

## Patient consent

For this type of publication informed consent was obtained from the patient.
